# Bacterial diversity in the jelly of shark Ampullae of Lorenzini: a holobiont perspective

**DOI:** 10.7717/peerj.20461

**Published:** 2026-01-05

**Authors:** Nataly Bolaño-Martínez, Benjamín Cristian Corona-Comunidad, Oscar Uriel Mendoza-Vargas, Luis E. Eguiarte, Valeria Souza

**Affiliations:** 1 Instituto de Ecología, Universidad Nacional Autónoma de México, Mexico City, Mexico City, Mexico; 2 Facultad de Estudios Superiores Zaragoza, Universidad Nacional Autónoma de México, Mexico City, México City, Mexico; 3 Facultad de Ciencias, Universidad Nacional Autónoma de México, Mexico City, Mexico City, Mexico; 4 Resercher, Océanos Vivientes A.C, Mexico City, Mexico City, Mexico; 5 Centro de Estudios del Cuaternario de Fuego, Fundación Cequa, Punta Arenas, Patagonia y Antártica, Chile

**Keywords:** Shark, Bacteria, Mexico, Jelly, Electrosensory system, Quorum sensing, 16S rRNA, Host-guest, Holobiont

## Abstract

The Ampullae of Lorenzini (AoL) are specialized electrosensory organs found in sharks and other chondrichthyans. They allow the detection of electric fields, temperature changes, and salinity variations. These organs contain a jelly composed of mucopolysaccharides, proteins, and ions, whose microbiota had not been previously characterized. In this study, we investigated and described the presence of bacteria associated with the AoL jelly in seven shark species from the Mexican coast, including three species from the family Sphyrnidae and four from the family Carcharhinidae. Bacteria present in the AoL jelly were cultured on selective media and characterized through 16S rRNA gene sequencing and phylogenetic analysis. We identified bacterial species belonging to the families Staphylococcaceae, Micrococcaceae, Bacillaceae, Vibrionaceae, Aeromonadaceae, and Microbacteriaceae. Additionally, we performed whole-genome sequencing of a subset of these bacterial isolates using the Illumina platform to identify genes related to AoL colonization and potential biological functions. We propose that the bacteria found in the jelly may be regular inhabitants of the AoL, as it provides the nutrients necessary for their persistence. This study represents the first report of bacteria associated with the AoL jelly in sharks, offering new insights into the microbiota of these organs and their potential influence on electrosensory function.

## Introduction

Chondrichthyans (Chondrichthyes) form a diverse group of cartilaginous fish that includes sharks, rays, and chimeras, with over a thousand species distributed globally (Weigmann, 2016). Among their most notable adaptations is the electrosensory system of the Ampullae of Lorenzini (AoL), a specialized extension of the lateral line widely distributed around the head and snout (Ebert, Fowler & Compagno, 2013). Each ampulla features a pore in direct contact with the aquatic environment, connected to an internal canal filled with a gelatinous material (henceforth referred to as “jelly” to distinguish it from the mucus found in other parts of the body of these fish), composed of mucopolysaccharides and proteins, which terminates in a sensory sac beneath the skin, closely connected to the nervous system (Doyle, 1963, 1967). This system enables the detection of weak electric fields produced by prey, variations in temperature and salinity (Sand, 1938), as well as navigation during oceanic migrations using the Earth’s magnetic field (Ebert, Fowler & Compagno, 2013).

The interest in understanding the composition of the jelly in the AoL date back to early anatomical and physiological studies (Lorenzini, 1678; Sand, 1938; Murray, 1962; Brown, 2003; Freitas et al., 2006). In *Squalus acanthias*, Jensen (1956) described mucopolysaccharides, hyaluronic acid, and chondroitin sulfate, while Doyle (1963, 1967, 1968) identified a sulfated polysaccharide composed of glucosamine, galactosamine, galactose, and uronic acid. In *Raja clavata*, urea was found to be part of the AoL (Murray & Potts, 1961), which is known to play an important role in the osmotic balance of fish (Smith, James & Herbert, 1929). Studies on rays (*Caliraja rhina*, *Raja binoculata*) and sharks (*Sphyrna tiburo*) revealed that keratan sulfate is the primary sulfated glycosaminoglycan, accounting for approximately 90% of the jelly’s polysaccharides, and is key in the reception and transmission of electrical signals (Josberger et al., 2016; Zhang et al., 2018). This hydrogel is primarily composed of water (approximately 97% of its weight) and marine ions such as Na^+^, Cl^−^, SO_4_^2−^, and K^+^, whose proportions confer high electrical conductivity, essential for detecting weak electric fields (Josberger et al., 2016). More recently, it has been proposed that chitin present in the jelly may act as a structural matrix that organizes the interaction between proteins and sulfated glycosaminoglycans, optimizing their electroconductive properties (Phillips et al., 2020).

Although the chemical and functional composition of the jelly in the AoL has been well documented, its microbiota remains largely unexplored. There are no prior reports on bacteria specifically associated with the AoL jelly, despite numerous studies identifying bacterial communities in other fish tissues, including the digestive tract (Aiso, Simidu & Hasuo, 1968), skin (Buck, Spotte & Gadbaw, 1984), lateral line (Crow, Brock & Kaiser, 1995), teeth (Interaminense et al., 2010), and mucus of elasmobranchs (Ritchie et al., 2017; Caballero et al., 2020). In particular, fish mucus has a composition similar to that of the AoL jelly, primarily consisting of water, salts, mucopolysaccharides, and glycoproteins such as mucins (Bansil & Turner, 2006). It is known to act as a physical, chemical, and biological barrier against environmental threats, particularly in environments with high microbial loads (Subramanian, MacKinnon & Ross, 2007; Subramanian, Ross & MacKinnon, 2008; Cone, 2009; Raj et al., 2011; Salinas, 2015). Mucins, in particular, are highly significant as they confer mucoadhesive, hydrophobicity, and elasticity to the mucus (Bansil & Turner, 2006), properties that are essential for the innate immune response, enabling the mucus to protect epithelial surfaces from chemical, enzymatic, and mechanical aggressions (Bansil & Turner, 2006; Dhanisha et al., 2018).

Fish mucus is part of the innate immunity associated with mucosal tissues (MALT), which includes structures such as the gut-associated lymphoid tissue (GALT), skin-associated lymphoid tissue (SALT), gill-associated lymphoid tissue (GIALT), and nasopharynx-associated lymphoid tissue (NALT), strategically distributed in physiologically important and sensitive organs in contact with the environment (Salinas, 2015).

In fish, mucus also exhibits antimicrobial properties, promotes skin regeneration, and modulates host-microbe interactions, either preventing or facilitating bacterial adhesion depending on its physicochemical characteristics and those of the microorganisms (Cameron & Endean, 1973; Al-Hassan et al., 1985; Hansen & Olafsen, 1999). Some bacteria even stimulate the secretion of antimicrobial compounds, promoting microbial competition that limits pathogen colonization (Salminen et al., 2010; Caballero et al., 2020).

Given the unique composition of the jelly in the Ampullae of Lorenzini, its exposure to the aquatic environment, and its similarities to fish mucus, which hosts bacterial communities with immune and ecological roles, there is a clear need to explore the microbiota of this sensory system from a holobiont perspective. Our study represents the first documented report of bacteria in the AoL jelly of chondrichthyans. We analyzed the bacterial communities present in seven shark species from the Mexican coast, evaluated the chemical composition and conductivity of the jelly, and identified genes potentially involved in colonization processes, thus providing a new perspective on the microbial ecology of this unique sensory system.

## Survey methodology

This research focuses on the study of microorganisms found in the jelly of the canals of the AoL in shark heads from Mexican coasts.

The samples used in this study were obtained from sharks incidentally captured by traditional fishers in Mexico, who provided the specimens for scientific research purposes. This study did not involve the intentional capture or sacrifice of animals. Shark fishing in Mexico is regulated by the Official Mexican Standard (NOM) NOM-029-PESC-2006 and the General Law on Sustainable Fisheries and Aquaculture (LGPAS), published in the Official Gazette of the Federation on February 14, 2007 (NORMA Oficial Mexicana NOM-029-PESC-2006, 2007). The research was conducted in compliance with national and international ethical standards for the use of animals in research. Approval from the Institutional Animal Care and Use Committee was not required, as no direct experiments with live animals were performed.

A sampling protocol and methodology were designed, adjusted to economic constraints and based on prior knowledge of elasmobranchs and *Vibrio*. For bacterial culture, the selective TCBS medium (thiosulfate-citrate-bile salts-sucrose agar) was used, which is specific for isolating *Vibrio* and effective in marine studies for facilitating the identification and characterization of these microorganisms (Nicholls, Lee & Donovan, 1976; Pfeffer & Oliver, 2003).

The main objective of the study was to identify and characterize bacteria isolated from jelly samples from the AoL canals of eight shark species in Mexico. The study included 10 sharks obtained through artisanal fishing at various locations along the Mexican coast. In Puerto Madero (PM), Chiapas (14°42′24″N 92°24′22″W), two *Sphyrna lewini*, one *Carcharhinus falciformis*, one *C. limbatus*, and one *Rhizoprionodon longurio* were collected. In Río Huach (RH), Quintana Roo (18°25′24.08″N 87°45′52.33″W), three sharks were collected: two *C. perezi* and one *S. mokarran*. Additionally, two sharks were collected from the La Viga Market (LV), Mexico City (19°21′36″N 99°07′26″W), sourced from the Gulf of Mexico: one *S. tiburo* and one *C. limbatus*.

Once the sharks captured by artisanal fishing were landed, the heads provided by the fishers were separated from the bodies and cleaned with alcohol and sterile gauze to prevent contamination. Manual pressure was then applied around the pores, using sterile gloves, to extract the jelly from the canals. The jelly was collected with a heat-sterilized bacteriological loop and inoculated onto a Petri dish with TCBS medium. The extracted jelly was preserved in 2 ml cryovials with 80% glycerol, and additional samples of pure jelly were stored in empty sterile cryovials. All containers were kept at 4 °C for transport to the laboratory.

Samples extracted from the shark head collected at the La Viga Market were transported to a laboratory located just 18 km away, where the same protocol for jelly collection and bacterial isolation was applied. Bacterial cultures conducted in the field and laboratory were incubated at 28–30 °C. Samples from the cryovials were recultured in the laboratory to confirm the species cultured in the field, and the remaining jelly samples have been preserved at −80 °C.

### Phenotypic characterization of bacteria associated with AoL jelly

#### Scanning electron microscopy

Jelly samples from the AoL were processed without chemical fixation and classified into very small sizes (<100 μm) and larger sizes (>0.5 mm), which were sectioned into 1 mm blocks. They were dried at room temperature in a laminar flow chamber on slides to avoid structural distortions. Subsequently, they were mounted on aluminum stubs with double-sided carbon tape to ensure electrical conductivity. The samples were coated with an approximately 20 nm thick gold film using a fine ion coater (JEOL JFC-1100). Finally, they were analyzed using a scanning electron microscopy (SEM) (JEOL JSM-6360LV), allowing for a general and detailed visualization of the sample morphology at the Scanning Electron Microscopy Laboratory, Institute of Marine Sciences and Limnology (ICMyL), UNAM.

#### Gram staining

Biological samples collected from the AoL in sharks were prepared for microscopic analysis using Gram staining. Heat fixation was applied by drying the samples on slides through flaming to adhere the bacterial cells. Each staining reagent (crystal violet, iodine solution, decolorizing alcohol, and safranin) was applied for one minute and washed with distilled water. This process was conducted at the Institute of Biomedical Research and the L-404 chemistry laboratory of the Faculty of Higher Studies Zaragoza (FESZ), UNAM.

After staining, the samples were examined under an optical microscope at 40× magnification and then at 100× with immersion oil to enhance resolution. This method allowed for detailed observation of the bacteria present in the AoL jelly samples.

#### Bacterial isolation from AoL jelly

Each AoL jelly sample was spread on TCBS agar plates (thiosulfate-citrate-bile salts-sucrose agar), adding 500 µl of liquid LB (Luria-Bertani) broth and sterile glass beads to homogenize the sample on the Petri dish. The plates were incubated at 37 °C for 24–48 h. The plate was swirled to ensure even distribution of the sample in the medium. Pure colonies were isolated from the TCBS agar and inoculated into 15 ml Falcon tubes with LB broth. The cultures were incubated at 30 °C and 200 rpm for 24–48 h using a shaker. Each LB isolate was preserved in 2 ml cryovials with 80% glycerol at −80 °C.

#### DNA extraction, PCR amplification, and 16S rRNA sequencing

One of the study’s objectives was to identify and characterize bacteria isolated from specific AoL jelly samples in sharks. After obtaining 58 bacterial isolates (Table 1), DNA extraction was performed using the DNeasy Blood and Tissue Kit (Qiagen, Hilden, Germany), following the manufacturer’s instructions. PCR amplification of the 16S rRNA marker was carried out using the universal primers 27F (5′ AGAGTTTGATCMTGGCTCAG 3′) as the forward primer and 1492R (5′ TACGGYTACCTTGTTACGACTT 3′) as the reverse primer (Lane, 1991; Turner et al., 1999). A reaction mixture containing 100–110 ng of template DNA, 5× GoTaq Flexi green buffer (M891A), 2.5 mM MgCl_2_, 2 µl of each deoxynucleotide triphosphate (dNTP), 10 µM of each primer, and 5 U/µl of Promega Taq polymerase was used in a final volume of 50 µl. PCR conditions included an initial denaturation of 3 min at 94 °C, followed by 30 cycles of 1 min at 94 °C, 1 min at 52 °C, and 1 min at 72 °C, with a final extension at 72 °C for 5 min, followed by cooling to 4 °C. The PCR product was visualized on a 1% agarose gel under UV light after staining with ethidium bromide (Devereux, Haeberli & Smithies, 1984). Sanger sequencing was performed at Macrogen USA and the Molecular Biology Laboratory, Institute of Biology, National Autonomous University of Mexico (UNAM).

**Table 1:** Shark sampling zones and the bacterial species associated with each.

Site	Year	Shark/Sea water	Bacteria (16S rRNA)
**Puerto Madero**	**2013**	*Carcharhinus falciformis*	*Staphylococcus xylosus* (4)
**(Chiapas)**			
		*Carcharhinus limbatus*	*Staphylococcus warneri* (1)
		*Rhizoprionodon longurio*	*Staphylococcus xylosus* (4)
		*Sphyrna Lewini*	*Staphylococcus saprophyticus* (3)
			*Staphylococcus xylosus* (1) *Vibrio spp*.**
**Rio Huach**	**2016**	*Carcharhinus perezi*	*Exiguobacterium profundum* (1)
**(Quintana Roo)**			*Vibrio antiquarius* (1)
			*Vibrio harveyi* (8)
			*Vibrio owensii* (10)
			*Vibrio rotiferianus* (3)
		*Sphyrna mokarran*	*Vibrio alginolyticus* (3)
			*Vibrio antiquarius* (2)
			*Vibrio azureus* (1)
			*Vibrio diabolicus* (2)
			*Vibrio owensii* (3)
		Sea water*	*Vibrio harveyi* (3)
			*Vibrio hepatarius* (1)
			*Vibrio midae* (1)
			*Vibrio owensii* (2)
			*Vibrio panuliri* (1)
			*Vibrio rotiferianus* (3)
			*Vibrio sp*. (1)
		*Carcharhinus limbatus*	*Leucobacter muris* (1)
**Mercado la Viga**	**2016**		*Pseudomonas fragi* (1)
**(Golfo of México)**			*Staphylococcus xylosus* (4)
		*Sphyrna tiburo*	*Aeromonas salmonicida* (1)
			*Arthrobacter sp* (1)
			*Leucobacter albus* (1)
			*Leucobacter komagatae* (1)

**Notes:**

The number in parentheses ( ) indicates the sequence numbers for each bacterium.

* Indicate seawater samples.

** *Vibrio spp*.

The sequences’ bacteria are very short and were only identified at the genus level.

#### Phylogenetic analysis of 16S rRNA sequences

Sequence editing was performed using BioEdit (Hall, 1999) and the UGENE v48.1 program (Okonechnikov et al., 2023); specifically, the UGENE 1.9.8 console version was used for sequence editing. Each 16S rRNA sequence for the bacterial isolates was verified with BLAST available at http://www.ncbi.nlm.nih.gov for taxonomic assignment, comparing them with the best alignment matches. A multiple alignment of 16S rRNA sequences (~800 bp) was performed using CLUSTAL 2.1 Multiple Sequence Alignments (Larkin et al., 2007).

A phylogenetic tree of the 16S rRNA gene was constructed using the Maximum Likelihood Estimation (MLE) method (Saitou & Nei, 1987) with UGENE v48.1 (Okonechnikov et al., 2023), implementing the HKY85 substitution model, and edited with FigTree version 1.42 (Rambaut, 2023).

#### DNA extraction, genomic sequencing, assembly, and annotation

For whole-genome sequencing, eight bacterial isolates from the jelly within the AoL canals of *S. mokarran* and *C. perezi* sharks were selected. These two shark species were chosen because a larger volume of gelatinous material was available for experimental replication, and whole shark heads were stored under controlled conditions, helping to preserve tissue integrity and minimize the risk of post-mortem contamination.

Genomic DNA was extracted and purified from 1.5 ml of LB broth using the DNeasy Blood & Tissue Kit (Qiagen, Hilden, Germany), following the manufacturer’s instructions for each sample. The concentration and purity of the purified genomic DNA were assessed using a NanoDrop 2000 UV-Vis spectrophotometer (Thermo Scientific, Waltham, MA, USA) in terms of the A260/A280 purity ratio. Additionally, DNA integrity was evaluated through 2% agarose gel electrophoresis.

For whole-genome sequencing of each isolate, MiSeq 2 × 300 (Illumina, Inc., San Diego, CA, USA) was used for paired-end sequencing. Read quality was checked with FASTQC (Andrews, 2010); the minimum quality value was set at 25, and low-quality sequences were removed using fastq_quality_filter from the FASTX-Toolkit (Hannon, 2010). Genomes were assembled *de novo* with SPAdes v.3.10.1 (Bankevich et al., 2012). Assembly quality was evaluated with QUAST v4.5 (Gurevich et al., 2013).

In addition to taxonomic identification based on the 16S rRNA gene, genomes were classified using the iron uptake regulator gene (fur) for each genome with the online program furIOS-1.0 (Machado et al., 2017) at http://www.cbs.dtu.dk/services/furIOS-1.0/. Similarly, virulence and colonization factors were searched in the eight genomes by comparing their sequences with the Virulence Factor Database (VFDB) by Chen et al. (2016) on the VFanalyzer platform. The complete genome sequences of the eight *Vibrio* strains and one *Exiguobacterium* strain were deposited in GenBank under accession numbers SAMN42149893–SAMN42149899.

#### Analysis of the chemical composition of AoL jelly

To identify the chemical functional groups in the AoL jelly (*S. mokarran*), some samples were dried at room temperature for approximately 40 min, while others were initially treated with chloroform (CHCl_3_) to remove polar molecules and then dried at room temperature with sodium sulfate added. Thin layers of each jelly sample were prepared for Fourier Transform Infrared Spectroscopy (FT-IR) analysis using potassium bromide plates at the Spectroscopy Laboratory L-301, Faculty of Higher Studies Zaragoza (FESZ), UNAM. Peaks were identified using the Spectrum software and assigned based on values reported in the literature (Doyle, 1967, 1968; Bell, 1973; Longas & Breitweiser, 1991; Frost et al., 2002; Al-Saidi et al., 2012; Prabhakar, Jain & Singh, 2012; Josberger et al., 2016; Ji et al., 2020; Vaičiukynienė et al., 2020). Amide A (3300–3500), Lipids (2800–3000), Amide I (1600–1800), Amide II (1470–1570), Ammonium (1384–1389), Amide III (1236–1350), GAG (1250–1220), sugars (800–1120).

#### Molecular structure of AoL jelly

To determine the molecular chemical structure (*i.e*., how different atoms or ions are connected), jelly samples from the AoL were analyzed. X-ray diffraction (XRD) analysis of the AoL jelly was performed using a Siemens D5000 instrument (scanning speed of 0.1°/s, Cu tube, Kα radiation) at the X-ray Diffraction Laboratory L-402, Faculty of Higher Studies Cuautitlán (FESC), UNAM. This analysis allowed the determination of the crystalline/amorphous structure and relationship of the AoL jelly. Some jelly samples were air-dried, while others were dried at 100 °C to observe how different drying conditions affect the molecular structure of the jelly, resulting in thin polymer layers with a mass of 3 g per sample, suitable for analysis in the equipment and for obtaining diffractograms.

#### Measurement of the conductivity of the gel from the Ampullae of Lorenzini

To measure the conductivity of the gel from the AoL, samples were collected from the dorsal and ventral areas of the head of the shark *S. mokarran*. The gel samples were obtained by pressing the AoL pores with fingers, using sterile gloves, to extract the jelly from the canals. The samples were collected using a sterilized laboratory loop.

Approximately one g (1 g) of gel was diluted in 50 mL of distilled water, stirring manually to promote dissolution. The measuring tube was then calibrated to a final volume of 10 mL. The conductivity of both samples was measured using a conductometer (Conductronic PC18) at 25 °C.

## Results

### Bacterial diversity in the AoL of sharks

**Figure 1:**
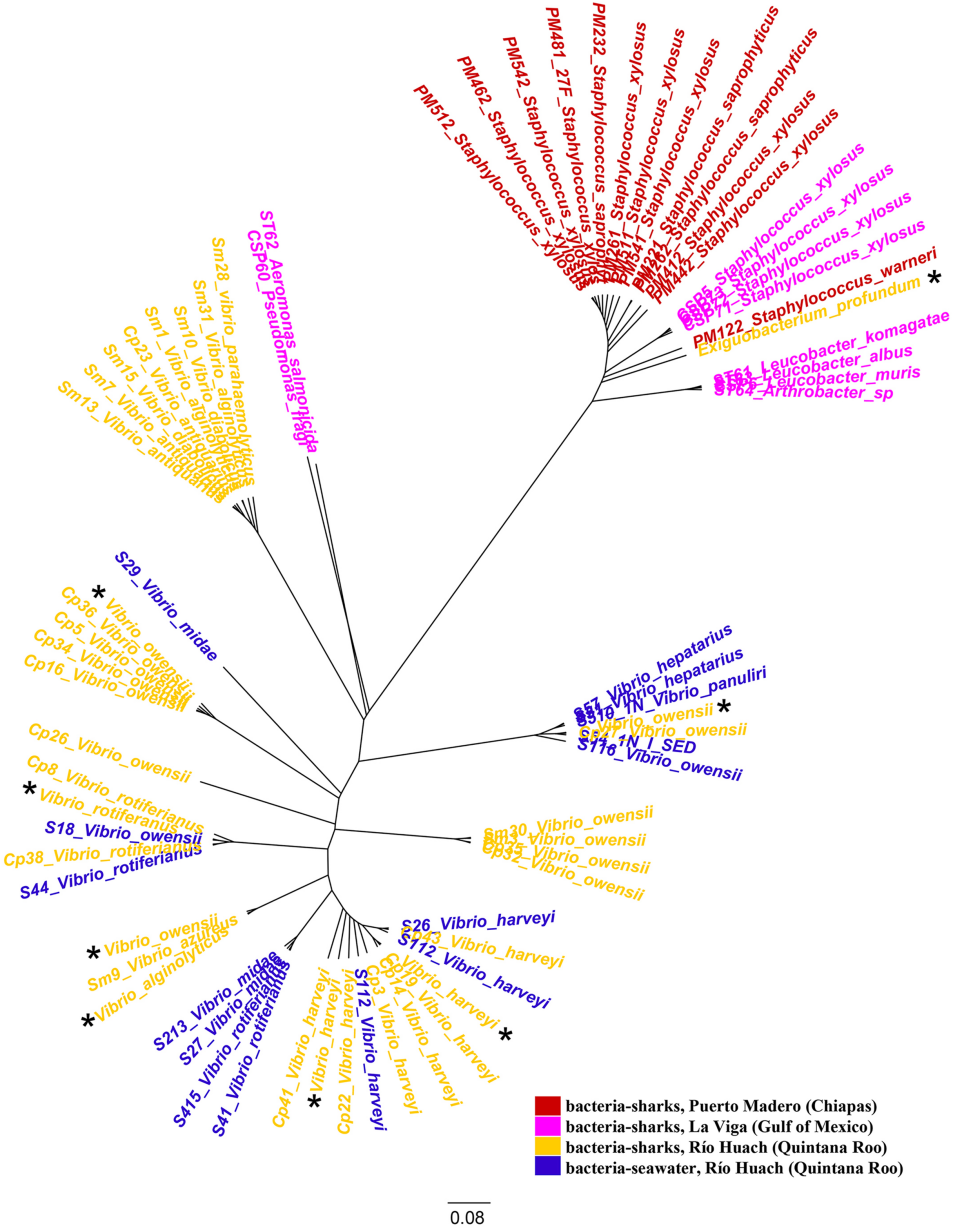
Phylogenetic tree of species based on partial 16S rRNA sequences, obtained using the Maximum Likelihood method. Bacteria associated with sharks captured in Puerto Madero (Chiapas) are shown in red, in La Viga (Gulf of Mexico) in pink, in Río Huach (Quintana Roo) in yellow, and free-living*Vibrio* from Río Huach, from seawater (Quintana Roo) in blue (Corona-Comunidad, 2023). The 16S rRNA sequences of the genomes are marked with a black asterisk.

We examined the jelly present in the AoL of seven shark species: *Sphyrna mokarran*, *S. lewini*, *S. tiburo*, *Rhizoprionodon longurio*, *Carcharhinus limbatus*, *C. falciformis*, and *C. perezi* (neonates, juveniles, and adults) from Mexican coasts. A total of 58 16S ribosomal RNA sequences and eight genomes (seven from the genus *Vibrio* (*V. alginolyticus* (1), *V. owensii* (3), *V. harveyi* (2), *V. rotiferianus* (1))) and one from the genus *Exiguobacterium* (*E. profundum* (1)) were obtained. From the bacterial strains we were able to culture, we identified 20 bacterial species belonging to several families: Staphylococcaceae, Micrococcaceae, Bacillaceae, Vibrionaceae, Aeromonadaceae, and Microbacteriaceae. The bacterial species identified in each shark are described in Table 1. It was observed that the majority of bacteria sampled from sharks in Quintana Roo belong to the family Vibrionaceae (33 out of 34 isolates), while in Puerto Madero, the majority belong to the family Staphylococcaceae (13 out of 13 isolates), and in La Viga, to the family Staphylococcaceae (four out of 10 isolates). The sequences of the bacteria and their associated environment are represented in the phylogenetic tree in Fig. 1.

### Phenotypic characterization of bacteria

**Figure 2:**
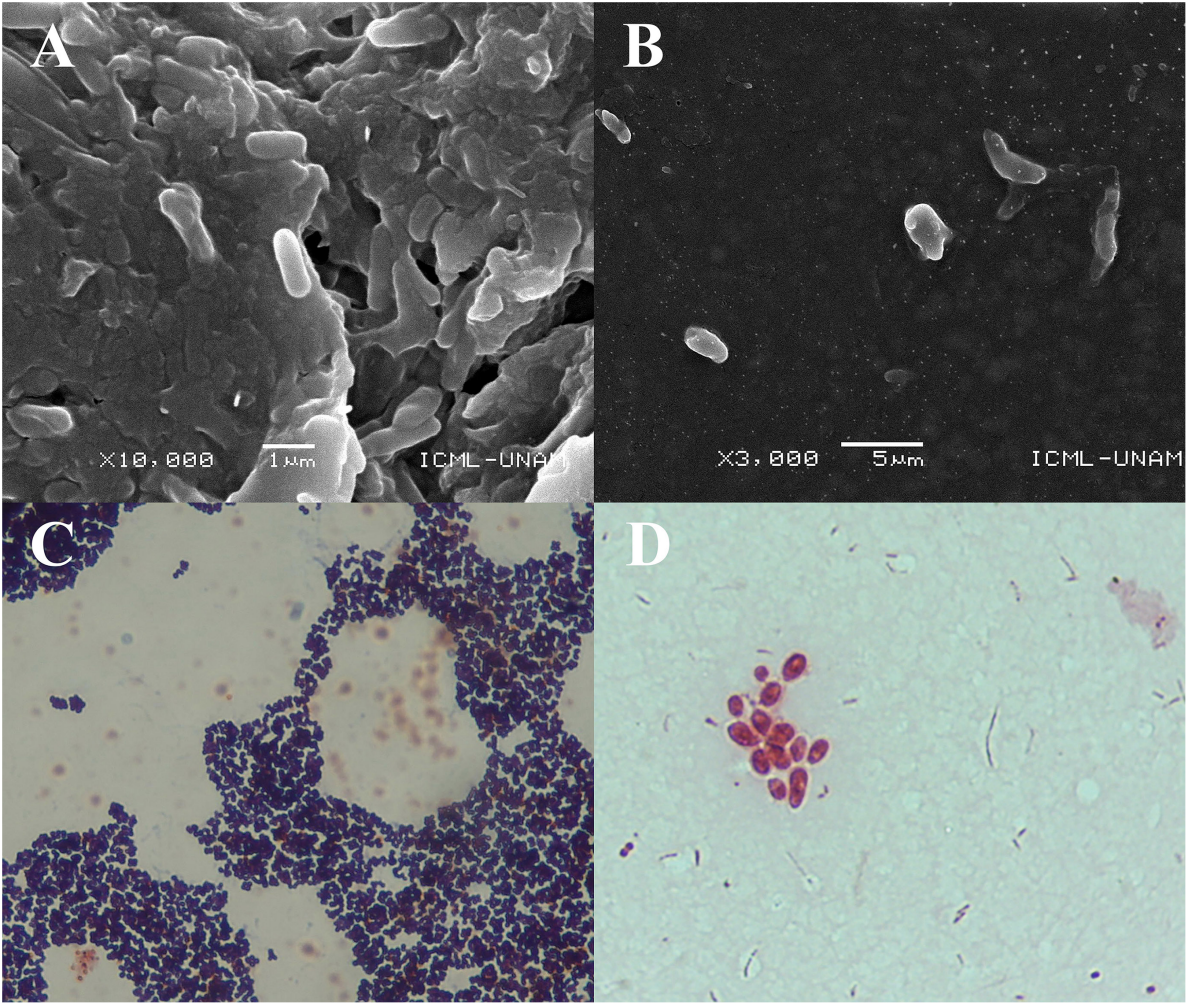
Images obtained by scanning electron microscopy (SEM) and staining Gram of gel samples from the Ampullae of Lorenzini (AoL) of the shark *Sphyrna mokarran*. The samples were processed without chemical fixation, air-dried at room temperature, mounted on aluminum stubs with carbon tape, and coated with a 20 nm layer of gold to enhance conductivity. (A) Image at 10,000× magnification, showing rod-shaped bacterial structures adhered to the gelatinous matrix. (B) Image at 3,000× magnification, showing dispersed bacteria within the gelatinous matrix, with a rod-shaped morphology and no visible aggregation. (C and D) images of bacteria obtained from the gelatin of the Ampullae of Lorenzini (AoL) of the sharks *Sphyrna lewini* (C) and *Sphyrna mokarran* (D), stained with Gram stain. C. Gram-positive bacteria with a rounded cocci shape, grouped in small clusters. The dark/purple colouration suggests they are Gram-positive bacteria, possibly from the *Staphylococcus* genus. (D) Gram-negative bacteria, showing short rods (cocco-bacilli) and some diplococci grouped in pairs or small clusters. The pink/red colouration indicates that they are Gram-negative bacteria, possibly from the genera *Vibrio*, *Neisseria*, or *Moraxella*. Magnification: 40× (2C) and 100× (2D).

The SEM images obtained at 10,000× magnification revealed cylindrical structures with morphological characteristics and dimensions compatible with rod-shaped bacteria (Figs. 2A and B). These structures were found adhered to the jelly matrix of the AoL. The arrangement of the bacterial structures and their preservation indicate the effectiveness of the non-invasive protocol used, allowing high-resolution imaging without compromising the integrity of the samples.

Microscopic observations revealed two predominant staining types in the analyzed samples. In one image (Fig. 2C), bacteria with purple staining were observed, indicative of Gram-positive bacteria. These bacteria retained crystal violet, suggesting a thicker and more complex cell wall structure characteristic of Gram-positive bacteria.

In another image (Fig. 2D), groups of rod-shaped bacteria with reddish staining were observed, indicating the presence of Gram-negative bacteria that did not retain crystal violet and were stained with safranin. The images show that both Gram-positive and Gram-negative bacteria were distributed within the jelly matrix of the AoL.

### Categories of genes related to colonization in shark AoL

The alignment of the seven genomes of *Vibrio alginolyticus*, *V. harveyi*, *V. rotiferianus*, and *V. owensii* against the VFDB database (Chen et al., 2016) identified 194 genes related to virulence and colonization (Table S1). Adhesion-related genes include msh/A-N, pil/A-D, LPS O-antigen (*P. aeruginosa*), nueB, and tadA. For antiphagocytosis, the genes are cps/A-J, rml/A, C, wbf/T, U, V, Y, wec/A, B, C, wza, wzb, and wzc. For chemotaxis and motility associated with flagellar machinery, the genes are che/A, B, R, V-Z, filM, fla/A, B, D, E, G, I, flg/A-N, flh/A, B, F, G, fli/A, D-S, flr/A, B, C, and mot/A, B, X, Y. Iron uptake-related genes are irgA, hut/A, R, vct/A, C, D, G, P, vibE, and sit/A, B, C, D. For quorum sensing detection, luxS and cqsA are present. Other genes related to bacterial secretion systems include eps/C, E-N, gspD, sycN, tyeA, vcr/D, G, H, R, V, vir/F, G, vop/B, D, N, Q, R, S, vsc/A, B, C, D, F-O, Q, R, S, T, U, X, Y, vxsC, hcp-2, vas/A-K, aaiI, T4SS effectors (Coxiella), and SCI-I T6SS (Escherichia). For toxins, the genes are tlh, aerA/act, cysC1, lgtF, and opsX/rfaC. The gene related to biofilm formation is adeG. For acid resistance, the genes are ure/B, G. Regarding serum resistance and immune evasion, genes associated with capsules (*Acinetobacter*), LOS (*Campylobacter*), and lipopolysaccharides (LPS) were identified, including rmlD and wbtL. Fimbrial adhesion determinant genes include stbA. The gene identified for encoding the α-enolase enzyme is eno. For genes associated with cell surface components, sugC was found, and in the “other” gene category, the O-antigen (Yersinia) and wcaG are included.

In the case of the alignment of the *Exiguobacterium profundu*m genome against the VFDB database (Chen et al., 2016), 41 genes were identified (Table S2). Virulence and colonization genes for adhesion are groEL, lap, plr/gapA, and tapT. For antiphagocytosis, rmlA/B, wecB, and capsule (*Enterococcus*). For cell surface components, sugC. For chemotaxis and motility, uge, fliI, and fliP. For copper uptake, ctpV. For enzymes, eno and an immune-inhibitory metalloprotease A (*Bacillus*). For iron uptake, viuC, fagB, hemL, and piaA. For secretion systems, fliQ, T6SS-II (*Klebsiella*), hlyIII, hemolysin III homolog (*Bacillus*), and hemolysin (*Clostridium*). For toxins, cylR2. For serum resistance and immune evasion, cap/F, O, wbt/B,E, fabZ, polysaccharide capsule (*Bacillus*), galU, and lgt. For intracellular survival, lplA1. For lipid and fatty acid metabolism, icl and panD. For phagosome detection, ndk. For regulation, csrA, cheY, and sigA/rpoV. Finally, for surface protein anchoring, lspA.

### Components of the AoL of sharks

#### Characterization of the jelly by Fourier transform infrared spectroscopy

**Figure 3:**
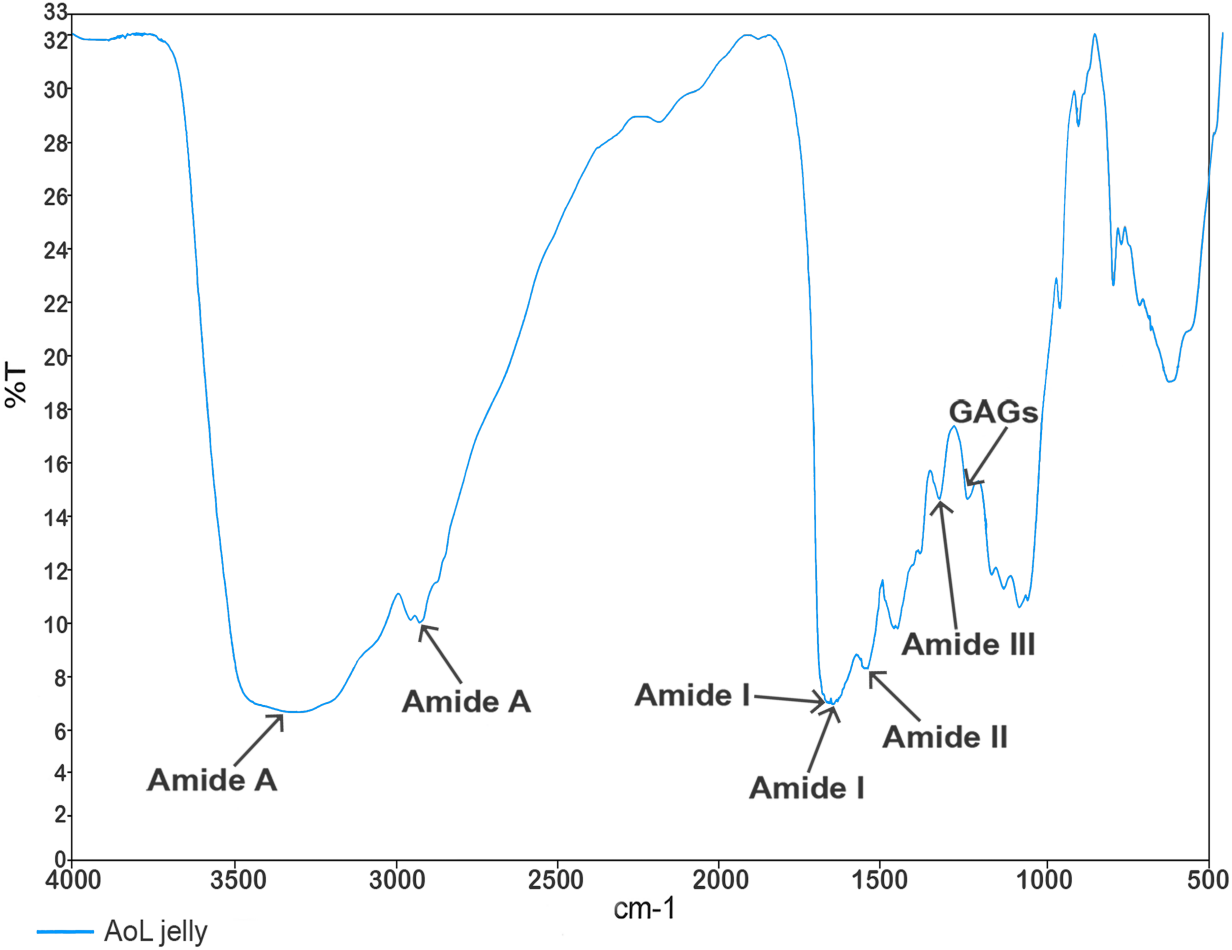
Characterisation spectrum of the AoL jelly by Fourier transform infrared spectroscopy (FTIR) of *S. mokarran* and *R*. *longurio*. Vertical axis: percentage of transmittance (%T); horizontal axis: wave number (cm^−1^).

In the analysis aimed at characterizing the components of the jelly present in the AoL of the studied sharks using Fourier transform infrared spectroscopy (FTIR) spectroscopy, values (Fig. 3) corresponding to absorption bands in the infrared spectra were obtained. These allowed the identification of signals associated with the following functional groups: Amide A at 3,305 and 2,932.65 cm^−1^, Amide I at 1,660.70 and 1,650 cm^−1^, Amide II at 1,541.07 cm^−1^, Amide III with a peak at 1,321.10 cm^−1^, and a peak corresponding to sulfated glycosaminoglycan (GAG) at 1,233.95 cm^−1^.

#### X-ray diffraction analysis of the jelly from the AoL of sharks

**Figure 4:**
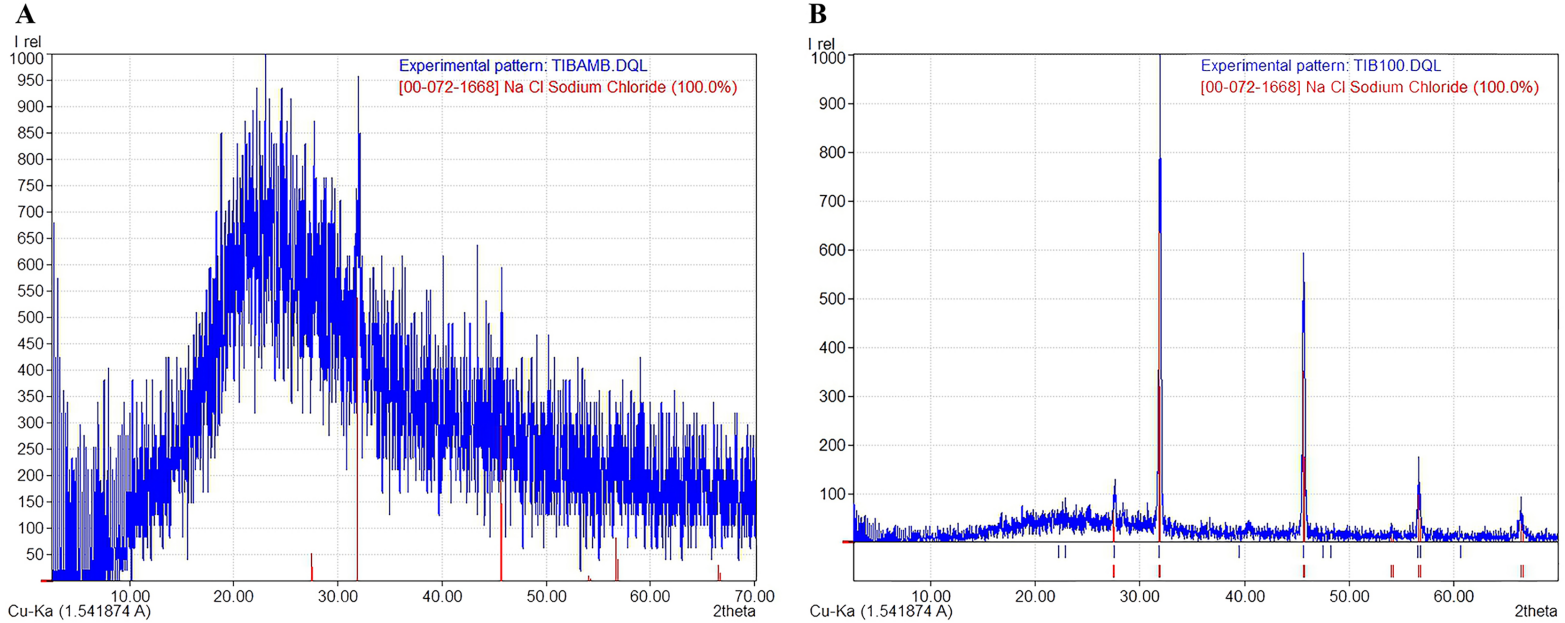
X-ray diffraction (XRD) patterns showing the amorphous and crystalline parts of the AoL jelly in samples. (A) Samples dried at room temperature. (B) Samples at 100 °C. The vertical axis represents the relative intensity (Irel) of the diffraction, and the horizontal axis represents the diffraction angle 2θ in degrees.

The X-ray diffraction (XRD) diffractograms obtained from the jelly samples were taken to help determine their structure and crystalline/amorphous relationships. The results indicated that samples dried at room temperature—to preserve the original molecular characteristics of the jelly in a state as close as possible to its natural condition—lacked a crystalline structure (Fig. 4A), and peaks corresponding to NaCl were identified, with a crystallinity of less than 3%, according to the International Centre for Diffraction Data (ICDD) card [00-072-1668], indicating a predominance of the amorphous phase in these samples. This amorphous signal was characterized by a broad hump rather than defined Bragg peaks, which is consistent with disordered, non-crystalline but flexible and cohesive biomolecular structures. In contrast, the samples dried at 100 °C (TIB100) showed a crystallinity rate of 51.40%, possibly associated with NaCl (Fig. 4B). The presence of NaCl in these samples could be due to salt concentration during dehydration, rather than being an intrinsic structural component of the jelly. The exact source of this crystallinity (jelly or NaCl) could not be conclusively determined. Finally, between diffraction angles of 1° to 35°, a curve corresponding to 48.60% of the amorphous portion of the AoL jelly was detected.

#### Conductivity of the AoL gelatin

In the dorsal area of the head, the conductivity measured 2.06 mS/cm, while in the ventral area, the conductivity was higher, reaching 2.36 mS/cm.

## Discussion

Although previous studies on elasmobranchs have reported genera such as *Vibrio*, *Photobacterium*, *Proteus*, *Pseudomonas*, *Citrobacter*, *Micrococcus*, *Staphylococcus*, and *Escherichia* in various organs (Buck, Spotte & Gadbaw, 1984; Grimes et al., 1985), and genera such as *Psychrobacter*, *Stenotrophomonas*, *Gordonia*, *Mycobacterium*, *Microbacterium*, *Caulobacter*, *Brevundimonas*, *Chryseobacterium*, *Nocardia*, *Bosea*, *Rhizobium*, *Delftia*, *Leucobacter, Acinetobacter*, *Exiguobacterium*, *Lysinibacillus*, and *Pseudoalteromonas* on the skin of rays (Ritchie et al., 2017), to our knowledge, no data exist on the microbiota of the AoL.

This research documents for the first time the bacterial diversity in the jelly of the AoL in sharks, expanding our understanding beyond their electroreceptive function.

### Phenotypic characterization of bacteria associated with AoL jelly

The SEM results clearly reveal cylindrical bacterial structures associated with the jelly of the AoL in sharks. These detailed SEM images highlight the preservation of bacterial structures, enabling future research into their roles in shark biology. The images allow for the identification of bacterial presence and morphology within the AoL jelly. Observing bacterial structures in this specific region suggests that microbial diversity may be adapted to the unique conditions of this organ.

Given the detailed insights from SEM regarding bacterial structures within the AoL of jelly, Figs. 2C and 2D, obtained through Gram staining, provide further insights into the microbial diversity within the gelatinous matrix of the AoL in sharks. The Gram staining indicates the coexistence of both Gram-positive and Gram-negative bacteria in this matrix, highlighting a microbial diversity that may include bacterial groups with potentially specialized functions. This observation aligns with previous studies demonstrating the coexistence of diverse microbial communities in specialized environments (Madigan et al., 2011; Garrity, 2007).

In Fig. 2C, the observation of rounded cocci with dark/purple staining indicates the presence of Gram-positive bacteria, likely from the genus *Staphylococcus*. This genus is well-documented for its ability to thrive in marine environments and colonize biological surfaces (Gerken et al., 2021), such as those of the studied sharks. The observed morphology and clustering are consistent with previous studies linking *Staphylococcus spp*. to biofilm formation and their role in providing structural integrity to microbial communities (Paharik & Horswill, 2016).

In contrast, Fig. 2D identifies Gram-negative bacteria through their pink/red staining and morphologies such as short rods (coccobacilli) and diplococci. These bacteria may belong to genera such as *Vibrio*, *Neisseria*, or *Moraxella* (Parija, 2023). *Vibrio* species are known for their association with marine ecosystems and their potential role in bioluminescence (Dunlap et al., 2007; Pipes & Nishiguchi, 2022). Additionally, other rod-shaped bacteria, such as those in the genus *Pseudomonas*, have been documented as potentially involved in symbiotic functions (Dunlap et al., 2007; Madigan et al., 2011). Therefore, Figs. 2C and 2D underscore the importance of the AoL matrix as a niche for diverse bacterial communities. This study lays the groundwork for continued exploration of bacterial diversity in the AoL and whether these microorganisms influence the biology and behaviors of sharks, particularly in the context of electroreception.

### Bacteria found in the AoL and possible environmental origins

Our study aligns with previous findings in elasmobranch organs by reporting bacterial species from the genera *Vibrio*, *Staphylococcus*, *Exiguobacterium*, *Leucobacter*, and *Pseudomonas*, along with species from the genus *Arthrobacter*. The presence of *Staphylococcus* in sharks sampled from Puerto Madero (Chiapas) is consistent with its detection in the oral cavities of sharks, as reported by Interaminense et al. (2010), Unger et al. (2014), Karns (2017). In samples from La Viga Market, bacteria from the genus *Leucobacter* predominated, a genus also identified in the freshwater stingray *Dasyatis sabina* (Ritchie et al., 2017). In cultures obtained from the jelly of the AoL of sharks sampled in Río Huach (Quintana Roo), the genus *Vibrio* was predominant, a group commonly observed in various body parts of multiple shark and ray species (Crow, Brock & Kaiser, 1995; Blackburn et al., 2010; Ritchie et al., 2017; Storo Salinas et al., 2021). Similarly, water samples from the same location (Río Huach) primarily yielded free-living bacteria of the genus *Vibrio*.

The presence of the same bacterial taxa in both the AoL jelly of *Sphyrna mokarran* and the surrounding water at the Río Huach sampling site suggests that these microorganisms may have been acquired from the environment. This pattern is analogous to the Hawaiian bobtail squid *Euprymna scolopes*, which acquires its symbiotic bacterium *Vibrio fischeri* from the marine environment (Pipes & Nishiguchi, 2022). Additionally, Storo Salinas et al. (2021) investigated the microbiome (gills, teeth, skin, cloaca) of five shark species in South Florida, including nurse sharks (*Ginglymostoma cirratum)*, lemon sharks (*Negaprion brevirostris*), sandbar sharks (*C. plumbeus*), Caribbean reef sharks (*C. perezi*), and tiger sharks (*Galeocerdo cuvier*). They noted that some microbial taxa in the surrounding seawater overlapped with the microbial communities of the sharks. This observation is consistent with our findings in Río Huach, Quintana Roo, where we detected *Vibrio owensii* in both the AoL of *S. mokarran* and *C. perezi* and in seawater samples from the capture site. Another similarity with their study is the evidence that the overall composition of bacterial communities differed significantly among shark species. We also observed this, with each shark species exhibiting a distinct group of bacterial species (Table 1). However, our sampling is limited and potentially biased due to the use of a selective medium like TCBS. Despite methodological differences—our study employed bacterial cultures and 16S rRNA sequencing, while theirs used 16S rRNA metabarcoding—our results are comparable. Therefore, we plan to expand our study by incorporating 16S rRNA metabarcoding in future research to deepen our understanding of the composition and functions of these microbial communities. Similarly, other studies have also demonstrated host-specific bacterial associations, independent of environmental location differences (Knowlton & Rohwer, 2003; Taylor et al., 2004; Troll et al., 2010; Nyholm & McFall-Ngai, 2022).

### Chemical and structural composition of the AoL jelly and its possible relationship with the electrosensory system of sharks

Studies in rays and sharks suggest that the jelly of the AoL is chemically composed of a mixture of proteoglycans and proteins (Doyle, 1963, 1968; Josberger et al., 2016; Zhang et al., 2018). This is consistent with the spectra obtained in our study through FT-IR with AoL jelly samples, where we detected signals related to GAG sulfate and amides (A, I, II, and III). It is known that keratan sulfate is the main component of the AoL jelly and provides high protonic conductivity within the electrosensory system of rays (Zhang et al., 2018).

The AoL enables sharks to detect extremely subtle changes in electric fields, with a sensitivity that can reach up to 5 nV/cm (Camperi, Tricas & Brown, 2007). The jelly filling the channels of these structures acts as a key conductive medium, facilitating the transmission of electrical signals to specialized sensory cells known as electroreceptor cells (Zhang et al., 2018). This jelly is crucial for sharks to detect prey by perceiving muscle contractions and other electrical phenomena generated by nearby organisms (Kalmijn, 1966). Given its essential role in electroreception, studying the conductivity of the jelly is fundamental to understanding how it modulates electrical signals and how this modulation integrates with the electroreceptive physiology of sharks (Von der Emde, 2001; Josberger et al., 2016; Ebert & Fowler, 2021). Therefore, we decided to include the measurement of AoL jelly conductivity in our study. It is important to note that the conductivity measurements conducted on the AoL jelly were limited in number; thus, the obtained values should be considered preliminary. Nevertheless, these values indicated an average conductivity of 2.21 mS/cm, which is consistent with previous reports of 1.8 ± 0.9 mS/cm in ray jelly (Josberger et al., 2016) and 2.44 ± 0.42 mS/cm in biofilms containing approximately 30% *Geobacter*, a bacterial genus known for its ability to transfer electrons extracellularly, contributing to electrical conductivity in biofilms (Lee et al., 2016), which could be a matrix similar to the AoL jelly. Further studies with a larger number of replicates will be necessary to confirm these findings and assess possible variations between species or environmental conditions.

The structure of the gel in the AoL of sharks showed a crystalline/amorphous nature, similar to other polymers with similar characteristics, such as bacterial biofilms (MacLean et al., 2007; Mosharaf et al., 2018; Palacios-Duchicela, 2019) and Nafion, a synthetic polymer with high electrical conductivity (Lee et al., 2016).

The disordered arrangement of molecular chains in amorphous polymers, which can result from the grouping of linear or branched macromolecules or highly cross-linked structures, leads to irregular and unstable networks that confer viscoelastic properties (Benavente, 1997). This structural organization is particularly relevant in biological hydrogels, where both the chemical composition and the polymeric network architecture allow for continuous ionic transport. These characteristics provide mechanical flexibility (Cui et al., 2020), which may facilitate the high protonic conductivity observed between sulfate groups (KS) in the jelly present in the AoL (Josberger et al., 2016).

Although we did not specifically investigate the presence of urea in this study, it is known that the gel of the AoL in certain ray species contains high concentrations of urea (Murray & Potts, 1961), an essential osmoprotectant that prevents dehydration in the marine environment (Yancey & Somero, 1979). This compound, also present in the internal fluids of these organisms (Murray & Potts, 1961), serves as a nitrogen source for urease-positive bacteria (Defoirdt et al., 2017). In our study, the species *V. harveyi* possesses genes ureB and ureG that encode enzymes for urea hydrolysis (Park et al., 2000; Defoirdt et al., 2017). Bacterial hydrolysis of urea produces ammonia (NH_3_) and carbon dioxide (CO_2_), generating ammonium ions (NH_4_^+^) and bicarbonate (HCO_3_^-^) in aqueous solution (Krajewska, 2009), which raises the local pH and neutralizes the acidity of the microenvironment (Mobley, Island & Hausinger, 1995). This process increases ionic conductivity, which is crucial for electroreception in elasmobranchs (Kalmijn, 1971; Josberger et al., 2016). The ability of these bacteria to metabolize urea provides them with adaptive advantages, allowing stable colonization of the jelly and the use of urea as a nutrient. Furthermore, the release of ammonia and the increase in pH could favor the proliferation of other bacteria tolerant to these conditions, modulating the microbial dynamics of the jelly and potentially regulating its physicochemical properties, which could influence the sensitivity of the electrosensory system (Josberger et al., 2016; Defoirdt et al., 2017).

### Possible interactions between the AoL microbiota and sharks

Our findings point to a complex biological scenario between the studied sharks and the bacteria found in the jelly of their Ampullae of Lorenzini. For example, proteins homologous to fucose-binding lectins and serotransferrin-1 have been identified in the gel of the AoL of rays (Zhang et al., 2018), molecules typically involved in pathogen recognition and innate immune responses (Van der Strate et al., 2001; Farnaud & Evans, 2003; Viejo-Díaz, Andrés & Fierro, 2004). This raises the possibility that the bacteria present in the AoL may have developed mechanisms to evade or resist the host’s defenses, such as quorum sensing modulation and the use of secretion systems, strategies previously described in symbiotic bacteria of other organisms (Cai et al., 2022; Suria et al., 2022; Wan et al., 2023).

In this study, we identified genes associated with colonization and persistence functions (*e.g*., adhesion, anti-phagocytosis, chemotaxis, motility, iron acquisition, quorum sensing, secretion systems, and toxin production) in the genomes of *Vibrio* and *Exiguobacterium*. These factors could facilitate their establishment, survival, and possible proliferation within the AoL, as has been proposed for other bacterial symbionts (Speare et al., 2018; Hernández, 2021; Suria et al., 2022; Cai et al., 2022; Wan et al., 2023). However, further studies are needed to elucidate the functional role of these bacteria within the AoL of sharks, and it is still necessary to determine whether the types of interactions observed are strictly mutualistic, commensal, or more complex, as local microbes could become harmful depending on the environmental conditions within the host (Perry & Tan, 2023).

On the other hand, it has been shown that the jelly of the AoL contains nitro groups (-NO2) (Josberger et al., 2016), which could serve as a nitrogen source for bacteria such as *Vibrio* and *Aeromonas* (Macfarlane & Herbert, 1984). Both genera are involved in the DNRA pathway (dissimilatory nitrate reduction to ammonium) (Macfarlane & Herbert, 1984; Bonin, 1996; Kelso et al., 1997), also known as nitrate ammonification, a process that occurs under low oxygen conditions where nitrate (NO_3_^−^) is converted to ammonium (NH_4_^+^). If this process is occurring in the jelly, it could help retain nitrogen within the jelly of AoL, making it available for various bacterial metabolic processes. It is important to conduct targeted studies to determine whether these processes are indeed occurring. In this context, the jelly would act not only as a conductive medium but also as a nutrient-rich matrix that supports the metabolic sustainability of the associated microbiota, while potentially limiting the proliferation of opportunistic bacteria.

Furthermore, bacteria such as *Pseudomonas putida* and *Aeromonas* species are known for their nitrifying capacity (Rodríguez et al., 2017), meaning they can convert ammonia (NH_3_) or ammonium (NH_4_^+^) into nitrites (NO_2_^−^) and subsequently into nitrates (NO_3_^−^), potentially closing the local nitrogen cycle within the AoL jelly. In other words, the bacteria could play a crucial role in the nitrogen cycle within the jelly-filled channels of the AoL. However, further studies are needed to clarify the role these bacteria play in the possible recycling of nutrients.

Additionally, species of *Flavobacterium*, *Micrococcus*, *Aeromonas*, and *Vibrio* have been documented to exhibit high keratanase productivity (Kitamikado & Ito, 1979) in the presence of keratan sulfate (KS). This ability to denature and assimilate KS would confer a competitive advantage for survival and colonization in the AoL jelly by transforming the polymeric matrix into bioavailable nutrients. From this perspective, the initial relationship between the host and the bacteria could be commensal, where the degradation of KS—the main component of the AoL jelly in rays (Zhang et al., 2018)—contributes to the stability of the microbial community without inducing pathogenicity. Moreover, there is a possibility that the relationship between the shark and its associated bacteria is mutualistic: the shark provides a nutrient-rich matrix through the jelly, and in return, the bacteria might help eliminate potential pathogens through their secretion systems. Although this is a plausible hypothesis, further studies are needed to confirm whether the bacteria identified in our study contribute to host defense.

It has been suggested that the epidermal mucus of elasmobranchs may have innate immune functions (Ritchie et al., 2017), which could explain the absence of wound-associated infections (Towner, Smale & Jewell, 2012; Chin, Mourier & Rummer, 2015). Specifically, the mucus of elasmobranchs may have a dual function: restricting and containing potentially pathogenic agents while simultaneously selecting specific bacteria to establish commensal or even mutualistic relationships, as described earlier. Therefore, the jelly of the AoL may complement the epithelial mucus by serving as an additional barrier to prevent the entry of pathogens into the shark’s bloodstream or internal organs. Furthermore, the biofilm formed by the microbial community selected by the shark may also contribute to protection against pathogens and enhance conductivity, thereby influencing the shark’s ability to detect electrical signals.

### Microenvironment of the AoL jelly and its relationship with the microbiota

The jelly found in the AoL of sharks, rich in mucopolysaccharides and proteins, constitutes a specialized microenvironment with abundant nutrients and unique physicochemical properties. These conditions could facilitate the colonization and establishment of stable bacterial communities, as suggested by the genes identified in this study. The bacterial species—or at least some of them—found in the AoL jelly might influence the electrosensory function of elasmobranchs through direct or indirect mechanisms: modulating immune responses, producing bioactive metabolites, or altering the chemical composition of the jelly.

Given the structural specialization of the jelly, we do not rule out the existence of a parallel microbial functional specialization, which would suggest processes of co-adaptation or even co-evolution between the host and its microbiota. Considering the similarities between the composition of the AoL jelly and the skin mucus of bony fish, we propose that the AoL may function as a specialized epithelial interface equipped with microbial selection mechanisms capable of promoting commensal or mutualistic relationships that benefit both the bacteria and the host.

The presence of bacteria in the jelly of the AoL raises intriguing questions about their possible role in the physiology of sharks. In this study, we found bacteria associated with the jelly in each of the AoL channels, leading us to wonder whether there might be a relationship between these microbial communities and the electrosensory system of elasmobranchs. Although there is currently no direct evidence confirming that these bacteria participate in electroreception or modulate electrical signals, their consistent presence in each AoL channel invites further investigation. Since the jelly facilitates the detection of weak electric fields (Bennett, 1971), it is plausible that the physicochemical properties of this gelatinous medium—potentially influenced or maintained by microbial activity—may affect its conductive capacity or contribute to the homeostasis of the electrosensory environment. Understanding these interactions is essential to advance our knowledge of the microbial ecology of the AoL and to explore whether the microbiota may play a previously unrecognized role in the sensory biology of sharks.

## Conclusions

In this study, we provide the first evidence of bacteria associated with the Ampullae of Lorenzini and, for the first time, present a preliminary characterization of this unique biopolymer, confirming the presence of sulfated glycosaminoglycans. We propose that the AoL channels could offer a refuge from the external environment, while the jelly might serve as a nutrient source for these bacteria. These findings open new possibilities for understanding the relationship between sharks and bacteria and highlight the need for further studies to confirm these findings and explore their ecological implications from a holobiont perspective.

## Supplemental Information

10.7717/peerj.20461/supp-1 Supplemental Information 1 Categories of Genes Related to Colonization in the AoL of Sharks present in the genomes of *Vibrio*. Categories of genes related to colonization in shark AoL jelly. Obtained from the alignment of seven *Vibrio* genomes (including the species *V. alginolyticus, V. harveyi, V. rotiferianus*, and *V. owensii*) against the VFDB database (Chen et al., 2016).

10.7717/peerj.20461/supp-2 Supplemental Information 2 Categories of Genes Related to Colonization in the AoL of Sharks present in the genomes of *Exigoubacterium*. Categories of genes related to colonization in shark AoL jelly. Obtained from the alignment of the *Exiguobacterium profundum* genome against the VFDB database (Chen et al., 2016).

10.7717/peerj.20461/supp-3 Supplemental Information 3 Genomes Vibrio and Exigoubacterium, Bolaño et al., 2024.

10.7717/peerj.20461/supp-4 Supplemental Information 4 The free-living (seawater)16S rRNA sequences. The free-living 16S rRNA sequences of Corona-Comunidad (2023).

## Additional Information and Declarations

### DNA Deposition

The following information was supplied regarding the deposition of DNA sequences:

The 16S rRNA sequences described are accessible *via* GenBank accession numbers PP967242 to PP967299.

The genome data described here are accessible *via* GenBank accessible *via* GenBank accession numbers SAMN42149892 to SAMN42149899 on PRJNA1129306.

### Data Availability

The following information was supplied regarding data availability:

The 16S rRNA sequences are available at GenBank PP967242 to PP967299.

The genome data are available at GenBank: SAMN42149892 to SAMN42149899; PRJNA1129306.
