# Supplementary material for: Bacterial diversity in the jelly of shark Ampullae of Lorenzini: a holobiont perspective
Source: PeerJ. 2026 Jan 5;14:e20461. doi: 10.7717/peerj.20461 (PMC12782034; doi:10.7717/peerj.20461)
Supplement: Supplemental Information 1 — Categories of genes related to colonization in shark AoL jelly. Obtained from the alignment of seven Vibrio genomes (including the species V. alginolyticus, V. harveyi, V. rotiferianus, and V. owensii) against the VFDB database (Chen et al., 2016). [file peerj-14-20461-s001.pdf]

Table S1. Categories of Vibrio Genes Related to Colonization in the AoL of Sharks

Virulence or colonization factors	Core genes of Vibrio	Accessory genes of Vibrio
Adherence	***msh/A,E,G,H,I,J,K,L,M,N***	***msh/B,C,D,F***
Adherence	***pil/B,C,D***	***pilA***
Adherence		***putative gene LPS O-antigen (P. aeruginosa)(Pseudomonas)***
Adherence		***nueB***
Adherence		***tadA***
Antiphagocytosis	***cps/A,C,F,G,H,***	***cps/B,D,E,J***
Antiphagocytosis	***cpsI***	***rml/A,C***
Antiphagocytosis		***wbf/T,U,V,Y***
Antiphagocytosis		***wbjD/wecB***
Antiphagocytosis		***wec/A,C***
Antiphagocytosis		***wza***
Antiphagocytosis		***wzb***
Antiphagocytosis		***wzc***
Chemotaxis and motility	***che,B,R,V,W***	***cheA***
Chemotaxis and motility	***che/Y,Z***	***flaD***
Chemotaxis and motility	***filM***	
Chemotaxis and motility	***fla/A,B,E,G,I***	
Chemotaxis and motility	***flg/A-N***	
Chemotaxis and motility	***flh/A,B,F,G***	
Chemotaxis and motility	***fli/A,D,E,F,G,H,I,J,K,L,N, O,P,Q,R,S***	
Chemotaxis and motility	***flr,A,B,C***	
Chemotaxis and motility	***mot/A,B,X,Y***	
Toxin	***tlh***	***aerA/act***
Toxin		***cysC1***
Toxin		***lgtF***
Toxin		***opsX/rfaC***
Biofilm formation		***adeG***
Cell surface components		***sugC***
Acid resistance		***Ure/B,G***

Continuation Table S1

Serum resistance and immune evasion		***Capsule(Acinetobacter)***
Serum resistance and immune evasion		***LOS(Campylobacter)***
Serum resistance and immune evasion		***rmlD***
Serum resistance and immune evastion		***wbtI***
Fimbrial adherence determinants		***stbA***
Iron uptake	***hutA***	***irgA***
Iron uptake	***vct/A,C,D,G,P***	***hutR***
Iron uptake		***vibE***
Iron uptake		***sit/A,B,C,D***
Quorum sensing	***luxS***	
Quorum sensing	***cqsA***	
Secretion system	***C,E,F,G,H,I,J,K,L,M,N***	***Undetermined***
Secretion system	***gspD***	***sycN***
Secretion system		***tyeA***
Secretion system		***vcr/D,G,H,R,V***
Secretion system		***vir/F,G***
Secretion system		***vop/B,D,N,Q,R,S***
Secretion system		***vsc/A-D,F-O,Q-U,X,Y***
Secretion system		***vxsC***
Secretion system		***hcp-2***
Secretion system		***vas/A-K***
Secretion system		***aaiI***
Secretion system		***T4SS effectors(Coxiella)***
Secretion system		***SCI-I T6SS(Escherichia)***
Enzyme		***eno***
Others		***O-antigen(Yersinia)***
