# Supplementary material for: Bacterial diversity in the jelly of shark Ampullae of Lorenzini: a holobiont perspective
Source: PeerJ. 2026 Jan 5;14:e20461. doi: 10.7717/peerj.20461 (PMC12782034; doi:10.7717/peerj.20461)
Supplement: Supplemental Information 2 — Categories of genes related to colonization in shark AoL jelly. Obtained from the alignment of the Exiguobacterium profundum genome against the VFDB database (Chen et al., 2016). [file peerj-14-20461-s002.pdf]

Table S2. Categories of *Exiguobacterium* Genes Related to Colonization in the AoL of Sharks

Virulence or colonization factors	Genes of Exiguobacterium
Adherence	groEL
Adherence	lap
Adherence	plr/gapA
Antiphagocytosis	rmlB
Antiphagocytosis	wbjD
Antiphagocytosis	Capsule(Enterococcus)
Chemotaxis and motility	uge
Chemotaxis and motility	fli/I,P
Enzyme	eno
Enzyme	Immune inhibitor A metalloproteinase(Bacillus)
Iron uptake	viuC
Iron uptake	fagB
Iron uptake	hemL
Iron uptake	piaA
Secretion system	fliQ
Secretion system	T6SS-II(Klebsiella)
Toxin	cylR2
Secretion system	hlyIII
Secretion system	Hemolysin III homolog(Bacillus)
Secretion system	Hemolysin(Clostridium)
Cell surface components	sugC
Copper uptake	ctpV
Immune evasion	cap/F,O
Immune evasion	wbt/B,E,I
Immune evasion	fabZ
Immune evasion	Polysaccharide capsule(Bacillus)
Immune evasion	galU
Intracellular survival	lplA1
Lipid and fatty acid metabolism	icl
Lipid and fatty acid metabolism	panD
Phagosome arresting	ndk
Regulation	csrA
Regulation	cheY
Regulation	sigA/rpoV
Serum resistance and immune evastion	lgt
Surface protein anchoring	lspA
